# Supplementary material for: Cloning, Expression Analysis, and Functional Characterization of Candidate Oxalate Transporter Genes of HbOT1 and HbOT2 from Rubber Tree (Hevea brasiliensis)
Source: Cells. 2022 Nov 27;11(23):3793. doi: 10.3390/cells11233793 (PMC9738940; doi:10.3390/cells11233793)

Supplementary Material S1: Bioinformatics online tools used in this research.

Name	Function	URL
TMPRED	Prediction of transmembrane domain	http://www.ch.embnet.org/software/TMPREDform.html
PSORT	Prediction of subcellular localization	https://www.genscript.com/psort.html
SignalIP	Prediction of signal peptides	http://www.cbs.dtu.dk/services/SignalIP
ProtScale	Analysis of amino acid affinity/ hydrophobicity	http://web.expasy.org/cgi-bin/protscale/protscale.pl
NCBI Conserved Domains	Prediction of conservative domains	https://www.ncbi.nlm.nih.gov/cdd
SMART		http://smart.embl-heidelberg.de/
NPS@SOPMA	Analysis of protein secondary structure	https://npsa-prabi.ibcp.fr/cgi-bin/npsa_automat.pl?page=npsa_sopma.html
I-TASSER	Construction of protein tertiary structure model	https://zhanggroup.org/I-TASSER/

Supplementary Material S2: Homology alignment and conserved domain information of FpOAR, HbOT1, HbOT2 and AtOT

1. Homology alignment of *FpOAR*, *HbOT1, HbOT2 and AtOT.*

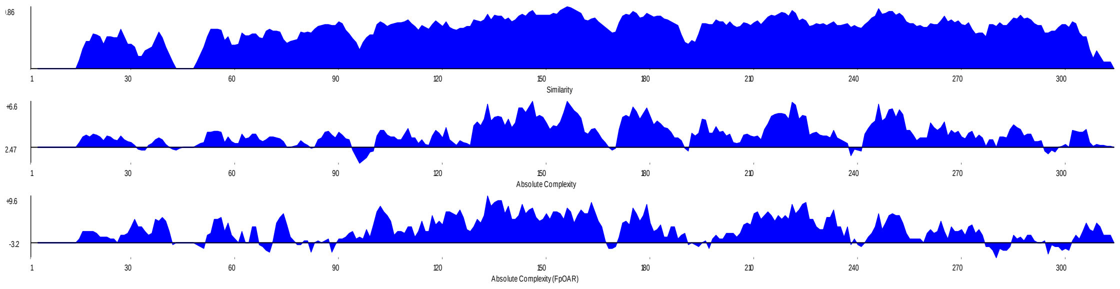

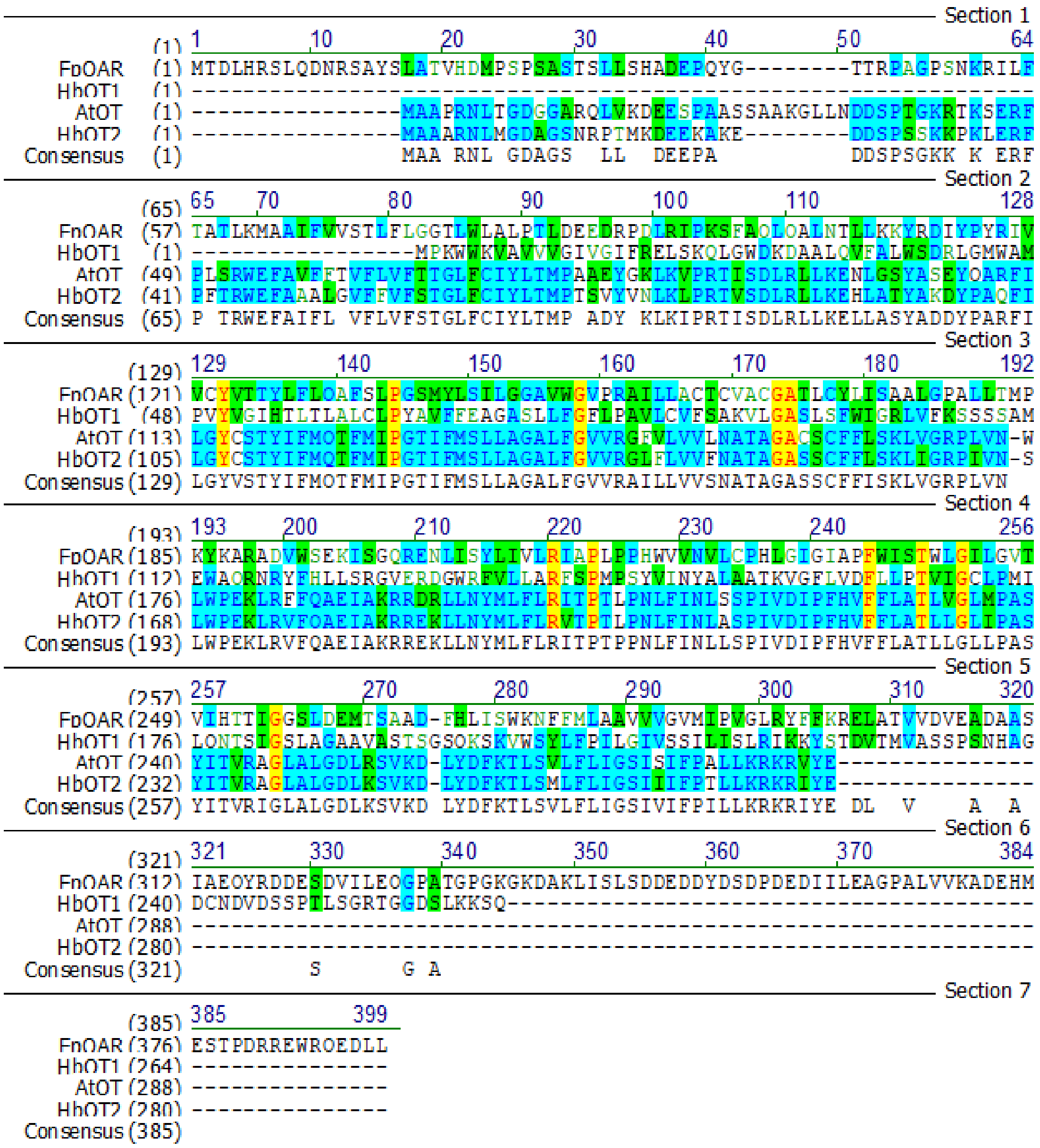

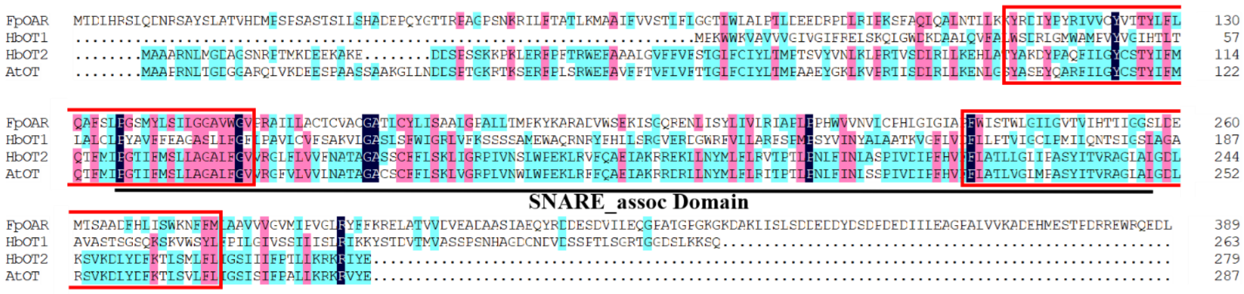

2. The SNARE-assoc conserve domain of FpOAR and homology blasted genes HbOT1, HbOT2 and AtOT

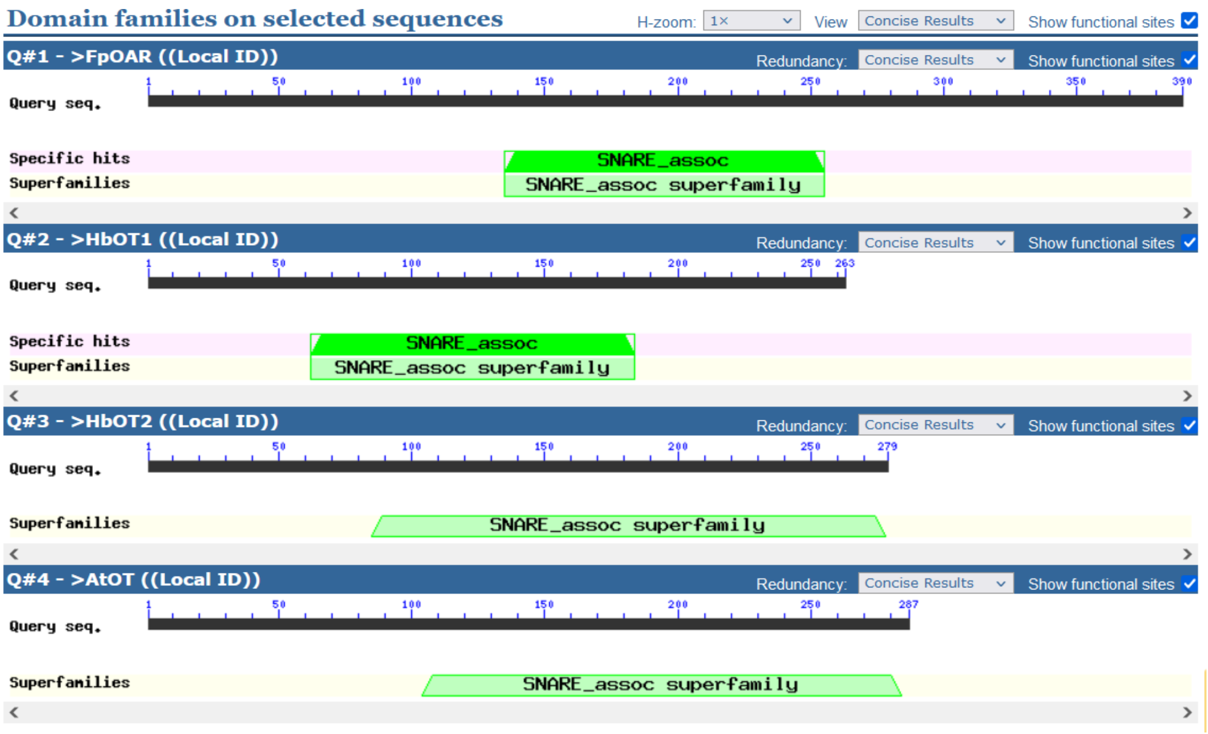

3. Similarity analysis of *FpOAR with HbOT1, HbOT2 and AtOT (A) and SNARE protein members from FpOAR, rubber trees and other plants.*

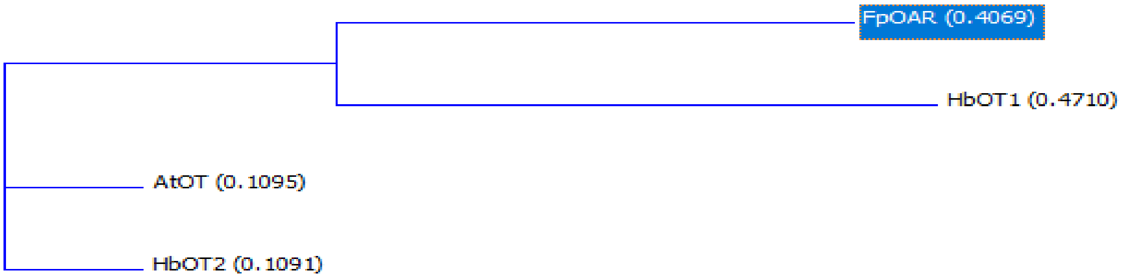

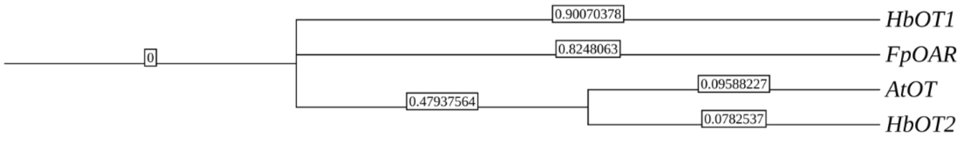

Supplement: Supplementary file 1 [file cells-11-03793-s001.zip › cells-1943529-supplementary.pdf]
